# Topical Spermidine Hyaluronate (Spd-HA) in Vulvovaginal Atrophy: A Preliminary Study

**DOI:** 10.3390/medicina61122104

**Published:** 2025-11-26

**Authors:** Irene Porcari, Michela Carena Maini, Carlo Angelo Ghisalberti, Caterina Tezze, Erica Trimarchi, Alessandra Graziottin, Mariachiara Bosco, Chiara Casprini, Simone Garzon, Stefano Uccella

**Affiliations:** 1 Department of Obstetrics and Gynecology, AOUI Verona, University of Verona, 37126 Verona, Italy; irene.porcari@gmail.com (I.P.); chiara.casprini96@gmail.com (C.C.); simone.garzon@univr.it (S.G.);; 2 Private Medical Practice, 20159 Milan, Italy; 3 Research Department, Tixupharma Srl, 20134 Milan, Italy; carlo.ghisalberti@gmail.com; 4 Department of Biomedical Sciences for Health, University of Milan, 20133 Milan, Italy; 5 Centre of Gynaecology and Medical Sexology, Department of Obstetrics and Gynaecology, San Raffaele Resnati Hospital, 20122 Milan, Italy; 6 Alessandra Graziottin Foundation for the Cure and Care of Pain in Women, NPO, 20122 Milan, Italy

**Keywords:** spermidine hyaluronate gel, vaginal health index, maturation index, maturation value, menopause, hypoestrogenic disorders, vulvovaginal atrophy, prasterone, estradiol, genitourinary syndrome of menopause (GSM)

## Abstract

*Background and Objectives*: Genitourinary syndrome of menopause (GSM), previously termed vulvovaginal atrophy (VVA), is a prevalent hypoestrogenic condition characterized by genital, sexual, and urinary symptoms. Although hormonal therapies are effective, many women are unwilling or unsuitable to use them. Spermidine hyaluronate (Spd-HA) has been reported to be effective in the treatment of vulvodynia and is currently under investigation for stress urinary incontinence. The aim of this study was to evaluate the efficacy of Spd-HA gel on the signs and symptoms of GSM. *Materials and Methods*: Five postmenopausal women with GSM presenting with vulvovaginal atrophy were enrolled in this prospective, single-arm, pilot study. They applied Spd-HA gel locally for eight weeks (three times/week for the first 4 weeks, followed by two times/week for the next 4 weeks). Vulvovaginal signs and symptoms were assessed at baseline (V1), 4 weeks (V2), and 8 weeks (V3) using the most bothersome symptoms (MBS), vaginal health index (VHI), and maturation index (MI) and value (MV). *Results*: Spd-HA gel was associated with MBS score improvement from baseline to week 8 [from (5.00 ± 2.00) to (1.60 ± 1.52)]. VHI score and MI improved from baseline to week 8. Compared to literature data, improvement in MVs obtained in women who received Spd-HA was greater than those obtained by hydrating products, but lower than those observed in women with estrogen or prasterone therapy. *Conclusions*: This preliminary experience suggests that Spd-HA gel ameliorates the vulvovaginal component of GSM. It may represent a promising non-hormonal option for women with GSM who are unwilling or unsuitable to receive hormonal therapy, warranting confirmation in larger controlled trials.

## 1. Introduction

Genitourinary syndrome of menopause (GSM), a term formally endorsed in 2014 by NAMS and ISSWSH, is now preferred over the historical term vulvovaginal atrophy (VVA), as it more accurately encompasses the genital, sexual, and urinary manifestations associated with the hypoestrogenic state of menopause [1,2,3,4].

GSM includes structural and functional changes involving the labia majora/minora, clitoris, vestibule, vagina, urethra, and bladder and may manifest with vaginal dryness, burning, dyspareunia, impaired sexual function, dysuria, recurrent urinary tract infections, stress urinary incontinence (SUI), urge urinary incontinence, and mixed urinary incontinence (MUI) [1,4].

For GSM diagnosis, these symptoms must be bothersome and should not be better accounted for by another diagnosis. GSM affects more than half of postmenopausal women and has a major impact on sexual health, interpersonal relationships, and quality of life [3].

A variety of treatment strategies are available to reduce GSM-related symptoms and to restore urogenital physiology. Treatment choice depends on symptom severity, coexisting medical conditions, and the patient’s wishes. First-line therapy for mild symptoms of GSM includes moisturizers and lubricants, while women with moderate to severe symptoms may benefit from pharmacological therapy [5]. Vaginal low-dose estrogen therapy or dehydroepiandrosterone (DHEA)—both approved by the Food and Drug Administration (FDA) and European Medicines Agency (EMA)—are the treatment of choice in these cases. Alternatively, oral therapy with selective estrogen receptor modulators (SERMs) can also be considered, as it is FDA- and EMA-approved [6]. Additionally, vaginal CO2 laser therapy has shown positive results as well [7]. However, a significant proportion of women cannot or prefer not to use hormonal therapy, particularly those with a history of hormone-sensitive cancers or contraindications, creating a growing need for safe and effective non-hormonal alternatives [8].

Recent clinical evidence highlights that several options are currently available for women seeking relief from GSM symptoms without using estrogen therapy. Vaginal dehydroepiandrosterone (DHEA, prasterone) has shown efficacy in improving vaginal cytology, pH, and sexual function [9,10,11]. However, as a hormonal precursor, DHEA remains contraindicated in women with a history of hormone-dependent cancers, limiting its use in a substantial subset of patients. Similarly, non-hormonal moisturizers and lubricants can provide symptomatic relief but do not restore epithelial trophism nor support long-term vaginal health [12].

In this context, recent clinical studies have provided evidence supporting non-hormonal and regenerative approaches as valuable first-line options for managing the vulvovaginal component of GSM, demonstrating significant improvements in symptoms, epithelial health, and patient satisfaction [3,13,14,15].

Emerging approaches restore the epithelial integrity and vaginal elasticity through non-hormonal pathways [16]. These developments provide the scientific foundation for novel medical devices such as spermidine hyaluronate (Spd-HA), as a potentiated version of hyaluronic acid (HA)-based vaginal moisturizers.

Among non-hormonal strategies, formulations containing HA demonstrated consistent clinical efficacy and safety. HA is a naturally occurring glycosaminoglycan with strong hygroscopic and viscoelastic properties that contribute to mucosal hydration and repair. Multiple randomized controlled trials and meta-analyses have reported that intravaginal HA improves dryness, burning, itching, and dyspareunia while restoring vaginal pH and epithelial integrity [17,18,19,20]. These results support HA as a first-line non-hormonal option in women with breast cancer or those unwilling to use hormones [21]. Several clinical studies, including those by De Seta et al. (2021) and Gustavino et al. (2021), further confirmed the beneficial effects of HA formulations in improving epithelial maturation, vaginal moisture, and comfort, thereby validating its use as an evidence-based non-hormonal medical device [14,15].

Recent advances have optimized HA formulations, including high-molecular-weight (HMW), cross-linked, and supramolecular complexes, which improve mucoadhesion, residence time, and water-retaining capacity [22,23]. These developments have shifted the role of HA products from simple moisturizers toward bioactive medical devices capable of promoting epithelial regeneration and barrier restoration.

Spermidine is a polyamine that has a pivotal role in many biological functions [24]. It exerts antioxidant and anti-aging effects via the autophagic process [25]. Importantly, the term “spermidine” was coined after its discovery in human semen along with spermine [26,27]. In physiological conditions, spermidine is prone to form supramolecular complexes (SMCs) [28]. Synthetic SMCs composed of spermidine and polyanionic polymers can trigger reparative processes in vaginal tissues through the enhancement of epithelial cell proliferation and tissue remodeling [29]. Beyond its structural and metabolic functions, spermidine activates autophagy, reduces oxidative stress, and enhances epithelial barrier stability [30,31,32]. These effects occur through surface-level cell interactions with limited invasiveness, while being associated with cytoprotection and mechano-stimulation, supporting its application in mucosal recovery and tissue homeostasis.

Spermidine hyaluronate (Spd-HA) gel (Ubigel donna™) is a registered medical product for genitourinary disorders, and clinical data on its use in women with localized provoked vulvodynia have recently been published [33]. The combination of spermidine and HA provides a dual mechanism: HA ensures hydration and film formation, while spermidine contributes by enhancing the mucosal resilience and by supporting regenerative processes in the epithelial and connective tissues. Early studies in vestibulodynia reported a clear symptom improvement with excellent local tolerability [24,34]. It has recently been proposed as a non-pharmacological medical device for women with mild GSM symptoms or for those who are not compliant or have contraindications to hormonal therapy. However, no studies have evaluated its use in women with GSM-related atrophy.

The aim of this study was to evaluate the efficacy of Spd-HA in reducing atrophic signs and to compare its effects with those reported for common moisturizers, vaginal estrogen (E2), or DHEA (prasterone) therapies.

## 2. Materials and Methods

### 2.1. Investigational Device

In this study, spermidine hyaluronate (Spd-HA) in gel—composed of high-molecular-weight hyaluronic acid (HMW-HA) and marketed with the commercial name Ubigel donna™ (Tixupharma Srl, Milan, Italy)—was used in agreement with the ISO 14155 guidelines on clinical practice [35].

### 2.2. Study Design and Participants

A pilot open-label, non-comparative, prospective clinical study was conducted.

It included women with a diagnosis of menopause (i.e., spontaneous amenorrhea of >12 months duration, spontaneous amenorrhea of >6 months duration associated with serum FSH concentrations > 40 IU/L, or iatrogenic menopause), with severe symptoms of VVA. Exclusion criteria were the following: vaginal infection, ongoing use of estrogen-progestin or progestin, ongoing use of vaginal products other than the investigational compound, previous laser therapy or vulvovaginal cryotherapy, diabetes mellitus, essential hypertension, tumors or chronic disease with life expectancy <2 years, autoimmune disorders, therapy with corticosteroids or immunomodulators in the last 8 weeks, therapy with antibiotics or antifungals in the last 7 days, history of drug and alcohol abuse, antidepressant therapy in the last 4 weeks, any allergy or intolerance to the components of the device, patient inability to comply with the protocol, and women who had already received the investigational device.

Women who met the inclusion criteria and agreed to study participation were instructed on the correct use of the vaginal gel, applied via cannula. The regimen consisted of local application 3 times per week for 4 weeks, followed by 2 times per week, for the next 4 weeks.

Participants were visited at baseline (visit 1, V1) and followed up at 4 weeks (visit 2, V2) and 8 weeks (visit 3, V3) after treatment initiation. At each visit, women were examined for signs of VVA and asked for VVA symptoms using the most bothersome symptom (MBS) score. Vaginal health index and degree of epithelialization through vaginal maturation index (MI) and maturation value (MV) were evaluated.

Given the small sample size, we performed a descriptive analysis without applying statistical tests.

### 2.3. Clinical Endpoints

- Most bothersome symptom (MBS) score, a self-reported questionnaire, was used to measure VVA symptom severity by assessing four key symptoms: dryness, itching, burning, and dyspareunia. The intensity of each of the four symptoms was rated on a 4-point ordinal scale (0 = absent, 1 = mild, 2 = moderate, 3 = severe). Dyspareunia was scored “severe” if sexual activity was not possible because of symptoms [36]. To assess the treatment’s overall subjective efficacy, a composite symptom severity score was calculated weekly, as the sum of the individual severity scores for the four symptoms (range 0–12).
- Modified vulvovaginal health index (VHI) is a merged version of the vaginal health index [37] and vulvar health index [38], which evaluate five components: vulvar appearance/color, vaginal inflammation, pain at speculum insertion, vaginal moisture, and epithelial integrity (Appendix A). A score from 1 to 5 is given to each component (score 1 is the lowest health status). The sum of the five components represents the total VHI score. A score ≤ 15 defines the presence of VVA. VHI was assessed at each visit (V1, V2 and V3).
- Maturation index (MI) is obtained by cytological examination and is the percentage of parabasal, intermediate, and superficial squamous cells/100 cells. Predominance of superficial cells indicates a normal trophic condition, whilst, vice versa, predominance of parabasal cells indicates a poor trophism, often associated with hypoestrogenism [39].
- Maturation value (MV) is calculated with the following formula: MV = % surface cells + (0.5 × % intermediate cells). A threshold of 40 defines vaginal atrophy [40].
- Potential adverse effects (PAEs) were recorded weekly.

### 2.4. Comparison with Other Treatments

We compared cytologic data (expressed as MV) after Spd-HA gel use to those reported in the literature after the use of a hydrating HA-based product [41]; 0.5% dehydroepiandrosterone (DHEA) cream (equivalent to 6.5 mg of intravaginal prasterone) [42]; and a low dose of estrogen (10 μg 17β-estradiol) [43]. For the selection of the comparison cohort, we chose cases from randomized studies [41,42,43] whose treatment lasted at least 8 weeks. Considering the heterogeneicity of hydrating vaginal products, an average value was used among HAs (5 mg in vaginal tablets) and polycarbophil-based moisturizers [44], which exhibit similar performance [23].

### 2.5. Ethical Standard

This study was conducted in agreement with the Declaration of Helsinki. Written informed consent was obtained from each participant before study initiation. The study was approved by the IRB of the AOUI Verona (3631CESC).

## 3. Results

### 3.1. Individual Case Reports

Five late postmenopausal women with severe symptoms of vulvovaginal atrophy were included in the study. As VVA represents the vaginal component of the broader genitourinary syndrome of menopause (GSM), all participants exhibited typical hypoestrogenic features of GSM. Detailed information regarding each specific case during the study is reported in the Supplementary Materials. The demographic characteristics of each patient are reported in Table 1, whereas the individual VHI data recorded at each visit are detailed in Table 2. 

### 3.2. Tolerability and Remarks

The product was generally well tolerated. In one case (Subject #2) the first treatment produced a transient vaginal itching which spontaneously resolved with no treatment. No further product-related adverse events were observed. An estimated good compliance was achieved in Subjects #1, #2, and #3. They agreed to be follow-up by phone calls after the end of therapy. They reported symptom remission for up to 2 months after the end of treatment. The link between poor compliance and cytologic outcomes (MI/MV) supports a possible observed treatment–effect correlation.

Noteworthy, poor compliance was recorded on Subjects #4 and #5 especially during the second cycle of treatment. A worsening pattern in cytology (MI, MV) might reflect the treatment discontinuation. Indeed, Subjects #4 and #5 refused follow-up.

### 3.3. Individual Subjective Patterns

**Figure 1:**
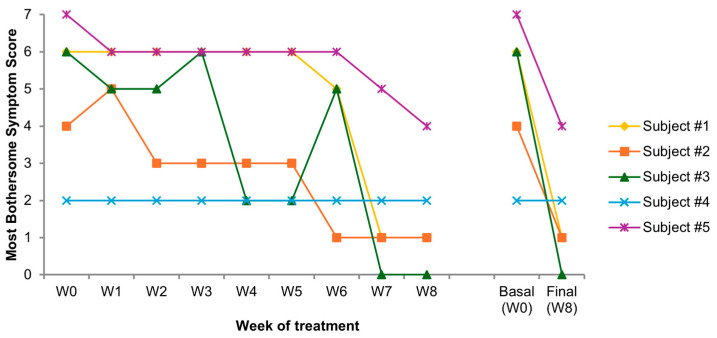
Individual most bothersome symptom (MBS) score from week 0 to 8 (W0–W8); trend from baseline (W0) to the end of treatment (W8).

Trend in individual MBS over the 8 weeks of treatment is shown in Figure 1.

### 3.4. Cumulative Subjective Outcome

**Figure 2:**
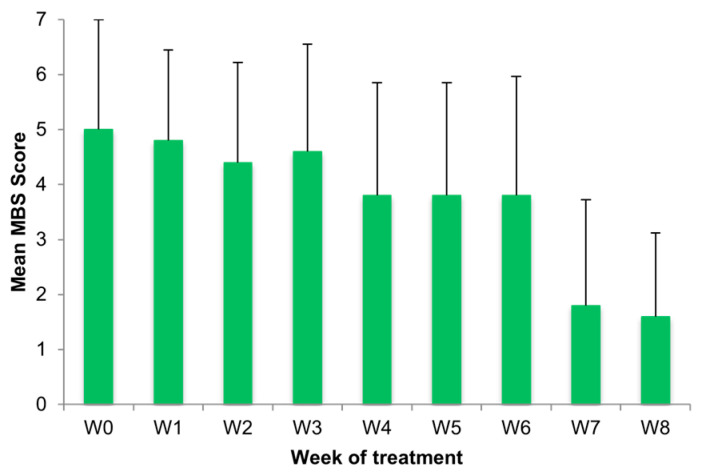
Mean MBS score from week 0 to 8 (W0–W8). There was a decrease in the mean MBS throughout the observation period, with the most important reduction between W6 and W7.

Throughout the observation period, there was a decrease in the mean MBS score, dropping from 5.00 ± 2.00 at V1 to 3.80 ± 2.05 at V2 and reaching a low of 1.60 ± 1.52 at V3 (Figure 2). Of note, as shown (Figure 2), the most important reduction in MBS was reported between week 6 (3.80 ± 2.17) and week 7 (1.80 ± 1.92).

### 3.5. Cumulative Objective Outcome

**Figure 3:**
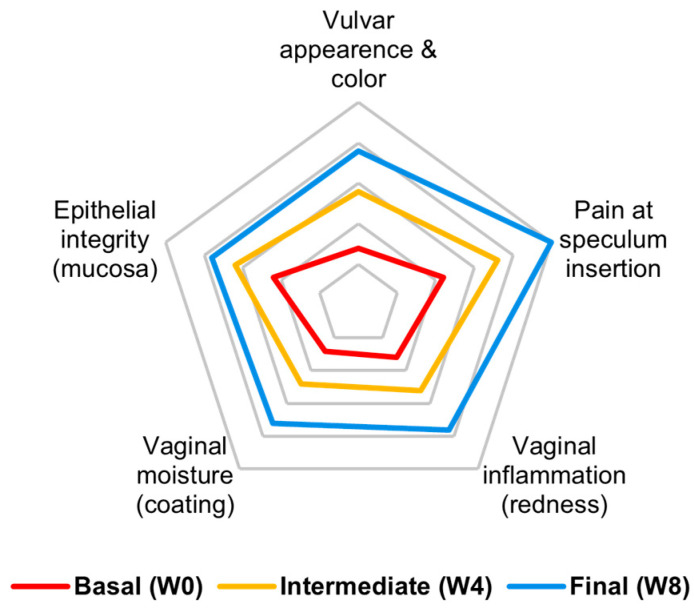
Vulvovaginal health index (VHI) components from baseline to visits 2 and 3 (cumulative participant score). There is a progression in every VHI item with a remission of introital pain, a linear improvement in vulvar and vaginal conditions and inflammatory status, and a partial restoration of lubrication. The graph represents the cumulative (mean) score of all participants for each VHI component.

VHI showed a remission of introital pain and a linear improvement in vulvar and vaginal conditions and inflammatory status, while lubrication was only partially restored. VHI showed a steadier pattern of recovery compared to subjective perception, which began to improve with a 3-week delay (Figure 3).

### 3.6. Individual Cytologic Data

**Figure 4:**
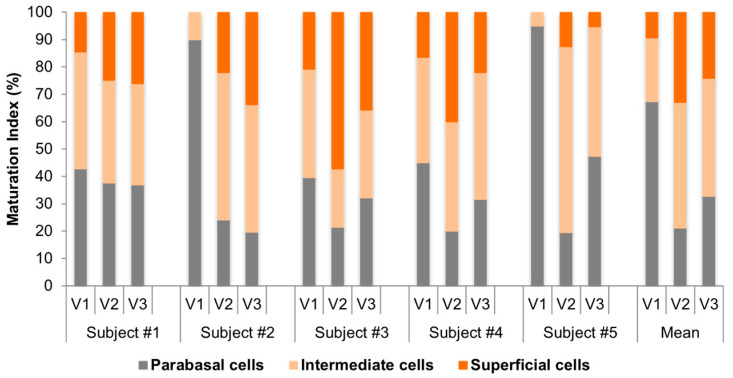
Individual changes in maturation index (MI) at V1, V2, and V3; mean value. The parabasal/intermediate/superficial cell ratio changed from 67/23/10 at V1 to 21/46/33 at V2 to 33/43/24 at V3. The decrease in parabasal and the increase in superficial epithelial cells indicate better estrogenization of the vaginal tissue (especially at V2).

The analysis of MI in the smears taken from the vagina before and after treatment showed a remarkable change in vaginal trophism. The parabasal/intermediate/superficial cell ratio changed from 67/23/10 at V1 to 21/46/33 at the V2 to 33/43/24 at V3 (Figure 4). MV improved from 32 at V1 to 47 at V3. Of note, this improvement was more relevant from V1 to V2 (32 to 53), while we observed a decrease in MV between V2 and V3 (53 to 47), which could be related to the reduced frequency of treatment prescribed from V2 to V3. This trend reflects the reduced frequency of application during the second phase of treatment.

### 3.7. Comparative Cytologic Data

**Figure 5:**
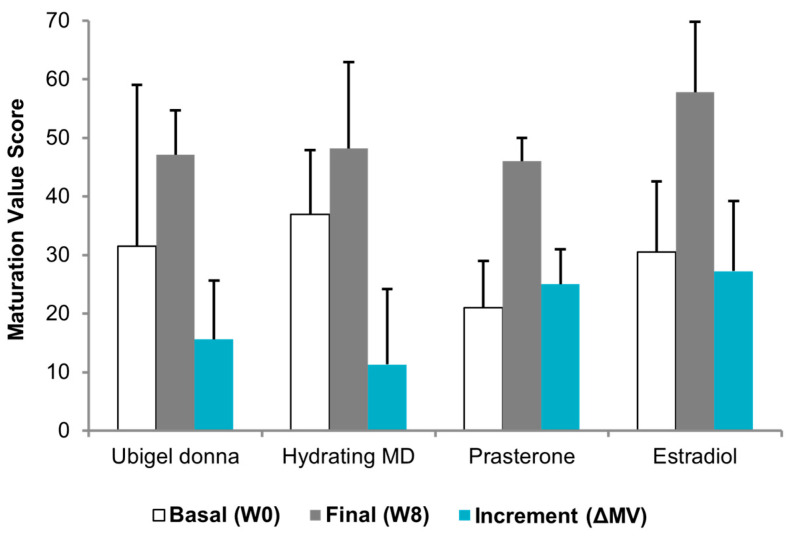
Maturation value (MV) at baseline (V1, white) and end-of-treatment (V3, gray) and incremental change (ΔMV, light blue). Spd-HA (*n* = 5) bars include mean ± SD and individual ΔMV (inset). Comparisons with hydrating medical device (MD), prasterone (DHEA), and estradiol (E2) are shown for descriptive context only; no between-treatment statistical testing was performed due to the pilot design and the use of historical cohorts.

Spd-HA gel provided an improvement in MI from 67/23/10 at V1 to 33/43/24 at V3 and prasterone from 59/40/1 to 14/78/8. E2 was associated with the highest MI improvement, from 42/54/4 to 4/78/18. The incremental difference in MV from baseline to V3 (ΔMV) was 15 (from 32 to 47) after spermidine hyaluronate therapy, 11 (from 37 to 48) after hydrating products therapy, 25 (from 21 to 46) after prasterone therapy, and 27 (from 31 to 58) after E2 therapy (Figure 5). For the Spd-HA cohort, the within-subject change in MV from V1 to V3 (ΔMV) was summarized as the mean ± SD and 95% CI; in this pilot sample, ΔMV showed a consistent improvement across participants. Between-treatment statistical testing versus historical cohorts was not performed, as such comparisons are not inferentially valid. Figure 5 therefore presents descriptive comparisons only.

## 4. Discussion

Our preliminary data show that Spd-HA gel is a promising therapy for GSM, allowing improvement in the parameters of genital health. GSM substantially affects women’s physical, psychological, sexual, and urinary well-being, and although highly prevalent after menopause, it is often normalized by both patients and clinicians as an inevitable consequence of aging. Several treatment options are available for the treatment of GSM-related symptoms, ranging from vaginal lubricants to hormonal topical vaginal applications. Extensive evidence exists on the efficacy of hormonal therapy for this condition [6,45]. However, many women are not willing to take hormonal compounds. Others have absolute or relative contraindications to them [46], such as women with previous and current hormone-dependent tumors (breast cancer or ovarian/endometrial adenocarcinomas) [47,48]. In these patients, treatments such as Spd-HA could become an important alternative for the management of GSM in postmenopausal women. Other non-hormonal options are embodied by electromagnetic methods, e.g., laser or radiofrequency therapies, which have yielded encouraging results [7].

**Figure 6:**
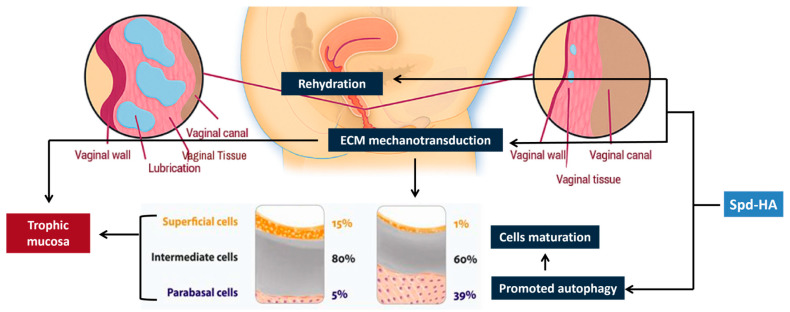
Mechanism of action of Spd-HA in VVA. It acts on vulvovaginal tissues: rehydration, antioxidant protection, biostimulation of the mechanotransducive elements within the extracellular matrix (ECM), and activation of autophagy.

The mechanism of action of Spd-HA is depicted in Figure 6.

Spd-HA gel can be considered a potentiated version of the HA-based medical devices commonly used in gynecology. While hydration remains a key factor in modifying the vulvovaginal microenvironment and eliciting maturation in atrophic tissues within GSM, other non-pharmacological modes of action seem to be at work. Indeed, the Spd-HA complex also activates cellular mechanotransducers on cell membranes, initiating force-induced conformations within cytoskeletal filaments [27,49,50,51]. The mechano-sensed event triggers a feedback system within the cytoskeletal network, activating contractile filaments and proteins (adherent and tight junctions) [52,53]. Mucosal reinforcement through dynamic activity at the cell periphery occurs through electrostatic interactions [54].

Furthermore, Spd-HA promotes antioxidant activity in the genitalia [51] and autophagy [32]. These ancillary effects, encompassing mechanotransduction combined with the anti-aging effects of autophagy [55], substantiate the clinical outcomes observed after Spd-HA treatment.

These mechanistic pathways are biologically plausible and supported by preclinical evidence. However, as expected for a small pilot study, the clinical data presented here are not intended to provide direct mechanistic support.

Preclinical evidence from an ovariectomized (OVX) rat model further supports the trophic potential of the Spd-HA complex [56]. In this study, local administration of Ubigel Donna™ was compared with hyaluronic acid (HA) and 17β-estradiol (E2) gels. The OVX model reproduces the hypoestrogenic condition of postmenopausal women, characterized by epithelial thinning, loss of superficial cells, and elevated vaginal pH. E2 treatment fully restored estrus-like epithelial morphology and endometrial thickness, and Spd-HA achieved a non-inferior histological recovery, with a significant increase in epithelial stratification, maturation index, and normalization of vaginal pH—effects not observed with HA alone. Notably, the maturation index after Spd-HA treatment was statistically non-inferior to that obtained with E2, demonstrating that the Spd-HA complex induces trophic and differentiating effects in the vaginal mucosa similar to those elicited by estrogen, yet without hormonal activity. These findings reinforce the regenerative and non-pharmacological mechanism of Spd-HA, in line with the clinical improvements observed in our postmenopausal cohort.

These outcomes are akin to the effect of estrogens on epithelial permeability and tissue health [57], yet they are not superimposable due to the lack of estrogenic activity. Importantly, Spd-HA’s biostimulation and anti-aging effects are entirely non-pharmacologic [58,59].

Recent clinical data on local Spd-HA therapy in women with vulvodynia showed efficacy in reducing pain and improving sexual function [33], but no studies on its use in postmenopausal women have been published to date.

In our study, we observed that Spd-HA, a non-hormonal therapy, effectively promoted a regenerative process in vaginal tissues and improved GSM symptoms involving the vulvovaginal compartment. Indeed, 8-week application of Spd-HA for women with moderate to severe GSM-related atrophy improved both subjective (MBS) and objective (VHI, MI, MV) scores. Vaginal cytology was selected as the endpoint for comparison with other topical options (HA lubricants, DHEA and estrogen therapy) due to its lower susceptibility to bias. With the limitation of every comparison with historical cohorts, Spd-HA showed superiority to non-hormonal lubricants in improving maturation value, although it was not superior to hormonal treatment. Notably, treatment adherence appears to have influenced the observed outcomes, as participants with poor compliance showed less consistent improvements in cytological parameters (MI/MV). This reinforces the importance of adherence when interpreting the clinical effect of Spd-HA.

The present study represents an encouraging pilot experience showing the promising outcomes of Spd-HA in managing GSM, specifically its vulvovaginal atrophic component. Indeed, all evaluated parameters, subjective symptoms, and objective gynecologic examination or cytology showed converging changes toward improved vulvovaginal health within the spectrum of GSM.

This study has important limitations. First, the small sample size (*n* = 5) and single-arm, open-label design substantially limit statistical power and preclude causal inference. Second, descriptive comparisons with historical cohorts are vulnerable to selection, performance, and measurement biases. Third, the short follow-up prevents assessing the durability of response. Fourth, patient-reported measures (MBS) may be susceptible to expectation bias, although they were complemented by objective cytological endpoints (MI/MV). Accordingly, these results should be viewed as preliminary and hypothesis-generating, warranting confirmation in adequately powered, randomized controlled trials with blinded assessment and longer follow-up.

Nevertheless, this pilot experience provides valuable feasibility data and supports the rationale for further research on Spd-HA as a non-hormonal therapeutic approach for GSM, particularly for women who are unwilling or unsuitable to receive hormonal therapy.

## 5. Conclusions

In this pilot study, Spd-HA gel proved to be effective in reducing vulvovaginal atrophic changes within the spectrum of GSM. It may therefore represent a viable non-hormonal option for women who are unwilling or unsuitable to receive hormonal therapy. A regimen consisting of 3–4 applications per week during the first 4-week cycle, followed by 2–3 applications per week during the subsequent 4-week cycle, is recommended. These preliminary findings require confirmation in larger, controlled clinical trials to further establish efficacy and long-term outcomes.

## Figures and Tables

**Table 1:** Demographic and medical data of participants.

Subject N.	Age	Years from Menopause	N. of Pregnancies	Medical History
#1	63	11	1	Factor VII deficiency; medications: alendronic acid and colecalciferol, Ca and Vitamin D; no history of HRT.
#2	63	13	1	Insomnia treated with melatonin; mild smoker; under HRT for 10 years, abandoned.
#3	71	18	3	Celiac; SUI; recurrent infective cystitis; hysterectomized; no history of HRT.
#4	71	21	2	Recurrent infective cystitis; no history of HRT.
#5	66	11	1	Hypothyroidism treated with thyroxine; uterine fibromatosis; no history of HRT.

HRT: hormonal replacement therapy.

**Table 2:** Individual VHI recorded at visit 1 (V1, baseline W0), visit 2 (V2, intermediate W4) and visit 3 (V3, final W8).

	Subject #1			Subject #2			Subject #3			Subject #4			Subject #5		
	V1	V2	V3	V1	V2	V3	V1	V2	V3	V1	V2	V3	V1	V2	V3
Vulvar appearance and color	2	3	4	1	3	4	1	3	4	2	3	4	1	2	3
Pain at speculum insertion	2	3	5	1	2	5	3	5	5	4	5	5	1	3	5
Vaginal inflammation (redness)	1	2	3	1	1	3	3	4	5	2	3	4	1	3	4
Vaginal moisture (coating)	2	3	4	2	2	4	1	3	4	1	2	4	1	2	2
Epithelial integrity (mucosa)	3	3	4	2	3	3	3	4	4	2	3	4	1	3	4
Mean	2.0	2.8	4.0	1.4	2.2	3.8	2.2	3.8	4.4	2.2	3.2	4.2	1.0	2.6	3.6
SD	0.7	0.4	0.7	0.5	0.8	0.8	1.1	0.8	0.5	1.1	1.1	0.4	0.0	0.5	1.1

## Data Availability

The data presented in this study are available within the article and Supplementary Materials.

## Supplementary Materials

The following supporting information can be downloaded at https://www.mdpi.com/article/10.3390/medicina61122104/s1, Supplementary Material S1: Case Series Information.

## Abbreviations

The following abbreviations are used in this manuscript:

Abbreviation	Meaning
DHEA	Dehydroepiandrosterone
ECM	Extracellular Matrix
E2	Estradiol
EMA	European Medicines Agency
FDA	Food and Drug Administration
GSM	Genitourinary Syndrome of Menopause
HMW-HA	High Molecular Weight Hyaluronic Acid
HRT	Hormone Replacement Therapy
IRB	Institutional Review Board
ISO	International Organization for Standardization
MBS	Most Bothersome Symptom
MI	Maturation Index
MV	Maturation Value
SERM	Selective Estrogen Receptor Modulator
SMC	Supramolecular Complexes
SUI	Stress Urinary Incontinence
V1, V2, V3	Visit 1, Visit 2, Visit 3
VHI	Vaginal Health Index
VVA	Vulvovaginal Atrophy

## Appendix A

### Modified Vulvovaginal Health Index

**Table A1:** Modified vulvovaginal health index (VHI) was utilized for the study, encompassing five components: vulvar appearance/color, pain at speculum insertion, vaginal inflammation (redness), vaginal moisture (coating), and epithelial integrity (mucosa).

VHI SCORE	1	2	3	4	5
Vulvar appearance and color	Very pale, reduced size	Pale, smaller labia	Intermediate	Mostly rose-colored	Rosy and healthy
Pain at speculum insertion	Very strong	Strong	Moderate	Minimal	None
Vaginal inflammation (redness)	Vivid red with rashes	Very red and inflamed	Intermediate, minor spots	Fair to good	Excellent, redness absent
Vaginal moisture (coating)	None	Poor	Fair	Good	Excellent
Epithelial integrity (mucosa)	Petechiae, ulcerate mucosa	Bleeds with light contact	Not friable, thin epithelium	Almost fully trophic	Normal
