# Supplementary material for: Topical Spermidine Hyaluronate (Spd-HA) in Vulvovaginal Atrophy: A Preliminary Study
Source: Medicina (Kaunas). 2025 Nov 26;61(12):2104. doi: 10.3390/medicina61122104 (PMC12734423; doi:10.3390/medicina61122104)
Supplement: Supplementary file 1 [file medicina-61-02104-s001.zip › medicina-3937502-supplementary.pdf]

**Supplementary material.** Case series information.

**Subjects #1**

A 63-year-old caucasian woman presented with complaints symptoms of VVA and sexual abstinence in the past three years due to a suppression of libido. She has never used hormone replacement therapy (HRT). At V1, she presented with a pale vulvar mucosa, redness of the vulvar vestibule and and absence of moisture. The diameter of the vaginal introitus was reduced. She reported pain at speculum (size small) insertion while the vagina inspection revealed redness of the vaginal wall. The patient had no intercourse until V2. At V2, speculum insertion was less painful, and we observed a reduced redness of the vaginal wall. Vaginal dryness was still partially present. At V3 she reported no dryness, although only a slight dyspareunia was still reported, and her physical exam showed marked improvement of vulvar mucosa with no red spots at the level of the mucosa of vestibulum. Speculum was painless and it revealed a healthy colored, vaginal epithelium and overall trophic status. Libido improved starting from week 2. The patients reported a short-lasting burning during week 1 and 2. Tolerability after week 2 was optimal. The subject furthermore stated an improved libido.

**Subjects #2**

A 63-year-old caucasian woman in menopause for 13 years. The women attempted symptoms management with oral HRT which was interrupted 5 years before study enrolment. After HRT discontinuation, the patients complained constant pain, inflammation accompanied by redness, dryness, dyspareunia leading to a drastic reduction in sexual life. On physical examination at V1, she presented with a pale vulvar mucosa with white patches. She reported strong pain at speculum insertion due to a contracted pelvic floor musculature. Internal examination was difficult, revealing an atrophic, redness vaginal mucosa easily bleeding, with no lubrication. At V2, the patient reported a complete relief in itching and dryness, although a slight dyspareunia still remained, hence she had not resumed sexual activity. At her physical exam we observed a marked improvement of vulvar mucosa, it looked less pale. Speculum insertion was less painful with no contraction of pelvic floor muscles. Vaginal mucosa did not bleed upon contact and appeared less red. At physical exam during V3, vulvar mucosa had a pink color and speculum examination was painless. Vaginal mucosa appeared wet and bleeding-free.

**Subjects #3**

A 71-year-old caucasian woman in menopause for 18 years presented with severe dryness and dyspareunia. She had total hysterectomy after pregnancies. She complained long-standing stress incontinence, so she underwent bladder-neck suspension with partial success. From that time, she

complained recurrent infective cystitis. On physical examination at V1, she presented with a pale, thin and frale vulvar vestibular mucosa. A first-grade rectocele was present. At speculum inspection, vaginal mucosa was thin, pale and significantly dry. At V2, she reported complete dryness and dyspareunia relief and lubricant-free intercourses. Her physical exam showed improvement: vulvar mucosa looked rose-colored; speculum insertion was painless and at inspection, vaginal mucosa looked turgid, moist and rose-colored. At V3 her condition continued to improve, with no dryness and dyspareunia. On physical examination vulvar mucosa significantly improved: she presented wet and rosy. Speculum was inserted without complaints; vaginal walls had an improved color and an overall trophic aspect. The subject reported itching at week 1, then itching and light burning feeling appear occasionally.

**Subjects #4**

A 71-year-old caucasian woman presented with symptoms of vaginal atrophy experiencing vulvar pruritus and burning at rest, for this she avoided sexual intercourse. She also reported recurrent urinary tract infection. She has never used HRT. At V1 she presented redness and dryness of vulvar mucosa. Speculum introduction caused moderate pain. Vaginal mucosa appeared atrophic, flushed, prone to bleeding upon touching. At V2, the patient reported no improvement of the atrophic symptoms. On physical examination vulvar mucosa switched from red to rose color. At speculum inspection, no pain neither bleeding was experienced, and vaginal mucosa became less flushed. At V3, vaginal mucosa had better discoloration and humidity, and speculum inspection revealed some trophic improvement. Product tolerability was good, but the subject recorded no improvement in all perceived score. Noteworthy, a poor compliance is suspected.

**Subjects #5**

A 66-year-old caucasian woman in menopause for 11 years presented with severe vaginal dryness, severe dyspareunia and large bleeding at every intercourse. After an episode of heavy bleeding, she underwent transvaginal pelvic ultrasound, PAP test and diagnostic hysteroscopy. Malignant pathologies were excluded. She has never used HRT. She reported uterine fibromatosis and hypothyroidism. On physical examination at V1, she presented with paleness of vulvar mucosa. Particularly, at the right side of labia minora and inside at furcation, inflammatory spots and petechiae were observed. She reported strong pain at speculum insertion (small-size). At inspection, vaginal mucosa appeared bright red. At V2, the patient complained a persistence of dyspareunia, even though she reported no bleeding at intercourses. During the physical exam, vulva appeared less pale compared to V1. At speculum inspection, vagina showed reduced redness as well. At V3, she reported a significant improvement of VVA symptoms, only a slight dryness and dyspareunia still remained. There have been no more episodes of bleeding during intercourse. Patient abolished sexual-aid

lubricant. Upon clinical evaluation vulvar mucosa appeared with rosier color. Speculum insertion was pain-free, and vaginal mucosa improved in color and trophic condition.
